# PD-L1 strong expressions affect the clinical outcomes of osimertinib in treatment naïve advanced *EGFR*-mutant non-small cell lung cancer patients

**DOI:** 10.1038/s41598-022-13102-7

**Published:** 2022-06-13

**Authors:** Kuo-Hsuan Hsu, Jeng-Sen Tseng, Tsung-Ying Yang, Kun-Chieh Chen, Kang-Yi Su, Sung-Liang Yu, Jeremy J. W. Chen, Yen-Hsiang Huang, Gee-Chen Chang

**Affiliations:** 1 Division of Critical Care and Respiratory Therapy, Department of Internal Medicine, Taichung Veterans General Hospital, No. 1650, Sect. 4, Taiwan Boulevard, Taichung, 407 Taiwan; 2 Division of Chest Medicine, Department of Internal Medicine, Taichung Veterans General Hospital, No. 1650, Sect. 4, Taiwan Boulevard, Taichung, 407 Taiwan; 3 Institute of Biomedical Sciences, National Chung Hsing University, No. 145, Xingda Rd., South Dist., Taichung, 402 Taiwan; 4 Department of Post-Baccalaureate Medicine, College of Medicine, National Chung Hsing University, No. 145, Xingda Rd., South Dist., Taichung, 402 Taiwan; 5 Faculty of Medicine, School of Medicine, National Yang Ming Chiao Tung University, No. 155, Sect. 2, Linong St., Taipei, 112 Taiwan; 6 Department of Life Sciences, National Chung Hsing University, No. 145, Xingda Rd., South Dist., Taichung, 402 Taiwan; 7 Division of Pulmonary Medicine, Department of Internal Medicine, Chung Shan Medical University Hospital, No.110, Sec. 1, Jianguo N. Road, Taichung, 402 Taiwan; 8 School of Medicine, Chung Shan Medical University, No.110, Sec. 1, Jianguo N. Road, Taichung, 402 Taiwan; 9 Department of Applied Chemistry, National Chi Nan University, No.1, University Road., Puli Township, Nantou, 545 Taiwan; 10 Department of Clinical Laboratory Sciences and Medical Biotechnology, College of Medicine, National Taiwan University, No. 1, Sect. 1, Jen Ai Road, Taipei, 100 Taiwan; 11 Department of Laboratory Medicine, National Taiwan University Hospital, No. 7, Zhung-Shan South Road, Taipei, 100 Taiwan; 12 Graduate Institute of Clinical Medicine, College of Medicine, National Taiwan University, No. 1, Sect. 1, Jen Ai Road, Taipei, 100 Taiwan; 13 Center of Genomic and Precision Medicine, National Taiwan University, No. 2, Syu-jhou Road, Taipei, 100 Taiwan; 14 Institute of Medical Device and Imaging, College of Medicine, National Taiwan University, No. 1, Sect. 1, Jen Ai Road, Taipei, 100 Taiwan; 15 Graduate Institute of Pathology, College of Medicine, National Taiwan University, No. 1, Sect. 1, Jen Ai Road, Taipei, 100 Taiwan; 16 School of Medicine and Institute of Medicine, Chung Shan Medical University, No.110, Sec. 1, Jianguo N. Road, Taichung, 402 Taiwan

**Keywords:** Medical research, Molecular medicine, Oncology

## Abstract

The impact of strong Programmed Death-ligand 1 (PD-L1) expression on the clinical outcomes of osimertinib in treatment naïve advanced *Epidermal Growth Factor Receptor (EGFR)*-mutant Non-small Cell Lung Cancer (NSCLC) patients remains uncertain. We enrolled advanced NSCLC patients who harbored sensitizing *EGFR* mutation and were treated first-line with osimertinib between 2017 and 2021. The PD-L1 expression level was also tested. A total of 85 patients were included. The objective response rate to osimertinib was 78.9%, with the disease control rate being 90.8%. Median Progression-free Survival (PFS) was 22.1 months, while median Overall Survival (OS) was not reached (NR). Patients with the exon 19 deletion experienced better PFS than those with the exon 21 L858R mutation (NR vs 12.4 months, aHR 0.24 (95% CI, 0.10 to 0.57); *p *= 0.001). Seventy-one of these 85 patients had reported on their PD-L1 expression. Patients with a PD-L1 < 50% experienced longer PFS than patients with a PD-L1 ≧50% (26.5 vs 9.7 months, aHR 0.19 (95% CI, 0.06 to 0.67); *p* = 0.009). Additionally, patients with a PD-L1 < 50% experienced better OS than those with a PD-L1 ≧50% (NR vs 25.4 months, aHR 0.09 (95% CI, 0.01 to 0.70); p = 0.021). Strong expressions of PD-L1 in treatment naïve advanced *EGFR*-mutant NSCLC patients were associated with poor prognoses in those undergoing treatment with osimertinib as first-line therapy.

## Introduction

*Epidermal Growth Factor Receptor (EGFR)* mutation is a driver mutation gene found most commonly in Non-small Cell Lung Cancer (NSCLC) within the Asian region^1,2^. The development of EGFR-Tyrosine Kinase Inhibitors (TKIs) has dramatically changed the treatment strategy in advanced NSCLC patients harboring the *EGFR* mutation^3,4^. Clinical trials and meta-analyses have demonstrated that patients with advanced *EGFR*-mutant NSCLC who were undergoing first- and second-generation EGFR-TKI as first-line treatments, experienced better Progression-free Survival (PFS) and fewer adverse effects when compared with those undergoing platinum-based chemotherapy^5–8^.

Osimertinib, an irreversible, selective, third-generation, EGFR-TKI is used to counteract Threonine 790 Methionine (T790M), the resistance mechanism of first- and second-generation EGFR-TKIs^9^. A phase 3 trial, AURA 3, showed that osimertinib provided significantly longer PFS than standard platinum-based chemotherapy in patients with advanced T790M-positive NSCLC who showed an acquired resistance to first-line EGFR-TKI treatment^10^. The FLAURA study also reported osimertinib as offering both better PFS and Overall Survival (OS) when compared with first-generation EGFR-TKIs treatments for NSCLC patients with *EGFR* mutation in a first-line setting^11,12^. Currently, osimertinib is the standard first-line treatment for those patients with advanced NSCLC harboring the sensitized *EGFR* mutation.

Recently, the treatment of NSCLC is entering the era of Immune Checkpoint Inhibitors (ICIs). Previous clinical trials involving advanced NSCLC patients have shown that the immune checkpoint blockade of Programmed cell Death-1 (PD-1), as well as Programmed cell Death-ligand 1 (PD-L1) provided better clinical outcomes for both PFS and OS, and fewer adverse effects when compared with standard chemotherapy^13,14^. Despite being imperfect, PD-L1 expression levels in tumor cells, as reflected by Immunohistochemical (IHC) staining, are predictive biomarkers for clinical effects in anti-PD-1 and anti-PD-L1 therapies^15,16^. Soo et al., found that NSCLC patients with the *EGFR*-mutant had lower PD-L1-positive rates than patients with wild type *EGFR*^17^. *EGFR*-mutated NSCLC patients have lower response rates to anti-PD-1 and anti-PD-L1 checkpoint blockades. Additionally, a meta-analysis revealed that ICIs do not improve OS when compared with the application of docetaxel to advanced NSCLC with *EGFR* mutation^18,19^.

Current studies regarding the correlation of PD-L1 expression levels in *EGFR*-mutant NSCLC patients and the clinical outcomes of first- and second-generation EGFR-TKIs have reported that mostly lower expression levels of PD-L1 during pre-treatment can predict a better Objective Response Rate (ORR) and PFS for EGFR-TKIs^20,21^. Advanced *EGFR*-mutant patients with strong PD-L1 expressions, defined as Tumor Proportion Score (TPS) ≧50%, have a greater chance of showing de novo resistance to EGFR-TKIs than patients with PD-L1 < 50%^22,23^.

Although a number of investigators have discussed the association between PD-L1 expression levels and the clinical efficacy of EGFR-TKIs, few studies have focused on a third-generation EGFR-TKI, osimertinib. The post-hoc analysis of the FLAURA study showed that in untreated *EGFR*-mutated advanced NSCLC, both the ORR and PFS resulting from osimertinib are unaffected by PD-L1 expression status. However, the analysis only adopted PD-L1 1% as the cut-off value^24^. Therefore, we conducted the present study in order to investigate the impact of strong PD-L1 expression on the clinical outcomes of osimertinib in treatment naïve advanced *EGFR*-mutant NSCLC patients.

## Material and methods

### Study design and patients

This study was a retrospective, single-center, observational study performed at Taichung Veterans General Hospital (TCVGH) in Taiwan. The study was conducted ethically in accordance with the World Medical Association Declaration of Helsinki and was approved by the Institutional Review Board (IRB) of TCVGH, Taiwan (IRB No. CF12019). All patients provided written informed consent for genetic testing, as well as use of their clinical data.

For the study, lung cancer patients were enrolled between December 2017 and December 2021. The inclusion criteria for patients were: (a) a diagnosis of histologically and cytologically confirmed NSCLC, (b) recurrence of, or diagnosed with, stage IV lung cancer according to the 8th edition of the American Joint Committee for Cancer (AJCC) staging system, (c) activating *EGFR* mutation with exon 19 deletion or exon 21 L858R point mutation, and (d) use of osimertinib as their first-line treatment. Patients were excluded if they were diagnosed with *EGFR* mutations other than exon 19 deletion and L858R mutation, were receiving other systemic treatments prior to their use of osimertinib, or were diagnosed with another active malignancy. Computed tomography of the chest was performed every three months in order for patients to qualify for National Health Insurance reimbursement. Treatment response to osimertinib was evaluated by the Response Evaluation Criteria in Solid Tumors (Version 1.1)^25^.

We collected for analysis each patient’s demographic and clinical data, including age, gender, smoking status, Eastern Cooperative Oncology Group Performance Status (ECOG PS), clinical stage, condition of brain metastasis at baseline, *EGFR* mutation status at baseline, PD-L1 expression status, as well as their PFS and OS results in regards to osimertinib treatment. PFS was defined as the time from the first dose of osimertinib to progression of the disease or death, while OS was defined as the time measured from the first dose of osimertinib to death.

### PD-L1 expression and the *EGFR* mutation test

PD-L1 IHC expression levels of tumor cells were tested using the anti-PD-L1 monoclonal antibody from the Ventana SP263 kit (Ventana Medical Systems, Tucson, AZ, USA), or the Dako 22C3 pharmDx kit (Dako, Carpinteria, CA, USA). *EGFR* mutations of tumor tissues were assessed using either the cobas® EGFR Mutation Test v2 or Matrix-assisted Laser Desorption Ionization-time of Flight Mass Spectrometry (MALDI-TOF MS). The method used in MALDI-TOF MS was based upon our previous studies^2,26,27^. The detection procedure was in accordance with the user’s manual provided in the MassARRAY® System (Cat. No.10411, SEQUENIM, San Diego, CA acquired by Agena Bioscience, http://agenabio.com/, San Diego, CA in 2014). Extracted DNA was used in a series of biochemical reactions, including 40 cycles of PCR reaction; SAP (Shrimp Alkaline Phosphatase) treatment and 200 cycles of signal nucleotide extension reaction by using the iPLEX Pro® reagent kit containing Sequenase, an iPLEX Pro® reaction mixture, and home-designed probes. After cleaning up using SpectroClean Resin, samples were loaded onto the matrix of the SpectroCHIP by Nanodispenser (Matrix) and then analyzed with the Bruker Autoflex MALDI-TOF MS. Data were collected and analyzed with MassARRAYTyper (Version 4) software (Agena Bioscience).

### Statistical analyses

To assess the inter-group differences (PD-L1 ≧50% group and PD-L1 < 50% group) in patient characteristics and demographic data, we used the Fisher’s exact test for age, gender, smoking status, ECOG PS, clinical stage, condition of brain metastasis at baseline and *EGFR* mutation status at baseline. Survival curves for both PFS and OS were estimated using the Kaplan–Meier method. The Cox proportional hazard model was used to evaluate differences in survival times of PFS and OS. All statistical tests were performed using SPSS 23.0 software (SPSS Inc., Chicago, IL, USA). Two-tailed tests and *P* values < 0.05 were considered significant.

## Results

### Baseline clinical characteristics of patients with osimertinib as first-line EGFR-TKI treatment

In total, data from 85 recurrent and stage IV *EGFR*-mutant NSCLC patients being treated with osimertinib as first-line EGFR-TKI therapy were analyzed. Their baseline characteristics are shown in Table 1. The median age was 63 years, with 33 of them male (38.8%), and 58 (68.2%) having no history of smoking. ECOG PS was from 0 to 1 in 77 patients (90.1%). Thirty-two (37.6%) patients were in stage IVA, while 38 (44.8%) were in stage IVB. Fifteen (17.6%) patients experienced disease recurrence after surgery. Brain metastasis was noted at baseline in 27 (31.8%) patients, while 61 (71.8%) harbored *EGFR* exon 19 deletion, and 24 (28.2%) had exon 21 L858R point mutation. Concerning PD-L1 expression levels, 8 (9.4%) patients had a Tumor Proportion Score (TPS) ≧50%, while 27 (31.8%) had a TPS between 1 to 49%. Negative results of PD-L1 were found in 36 (42.4%) patients, with 14 (16.4%) having no PD-L1 report.

**Table 1:** Patients' characteristics and demographic data.

Characteristics	N = 85
Age (years), median (range)	63 (32–86)
**Gender, n (%)**	
Male	33 (38.8)
Female	52 (61.2)
**Smoking status, n (%)**	
Never-smokers	58 (68.2)
Former or current-smokers	27 (31.8)
**ECOG PS, n (%)**	
0–1	77 (90.1)
2–3	8 (9.9)
**Stage, n (%)**	
Post-operation recurrence	15 (17.6)
Stage 4A	32 (37.6)
Stage 4B	38 (44.8)
**Brain metastasis at baseline, n (%)**	
Yes	27 (31.8)
No	58 (68.2)
**Baseline *EGFR* mutation status, n (%)**	
Exon 19 deletion^#^	61 (71.8)
Exon 21 L858R^&^	24 (28.2)
**PD-L1 expression level, n (%)**	
Negative	36 (42.4)
1–49%	27 (31.8)
≧50%	8 (9.4)
Unknown	14 (16.4)
**Treatment response**	
Complete response, n (%)	1 (1.3)
Partial response, n (%)	59 (77.6)
Stable disease, n (%)	9 (11.9)
Disease progression, n (%)	7 (9.2)
Could not be evaluated, n	9
Objective response rate	78.9%
Disease control rate	90.8%

ECOG PS, Eastern Cooperative Oncology Group performance status; EGFR, epidermal growth factor receptor; PD-L1, Programmed death-ligand 1.

^#^1 Exon 19 deletion + Exon 20 insertion; ^&^1 Exon 21 L858R + T790M.

### The clinical efficacy of osimertinib

**Figure 1:**
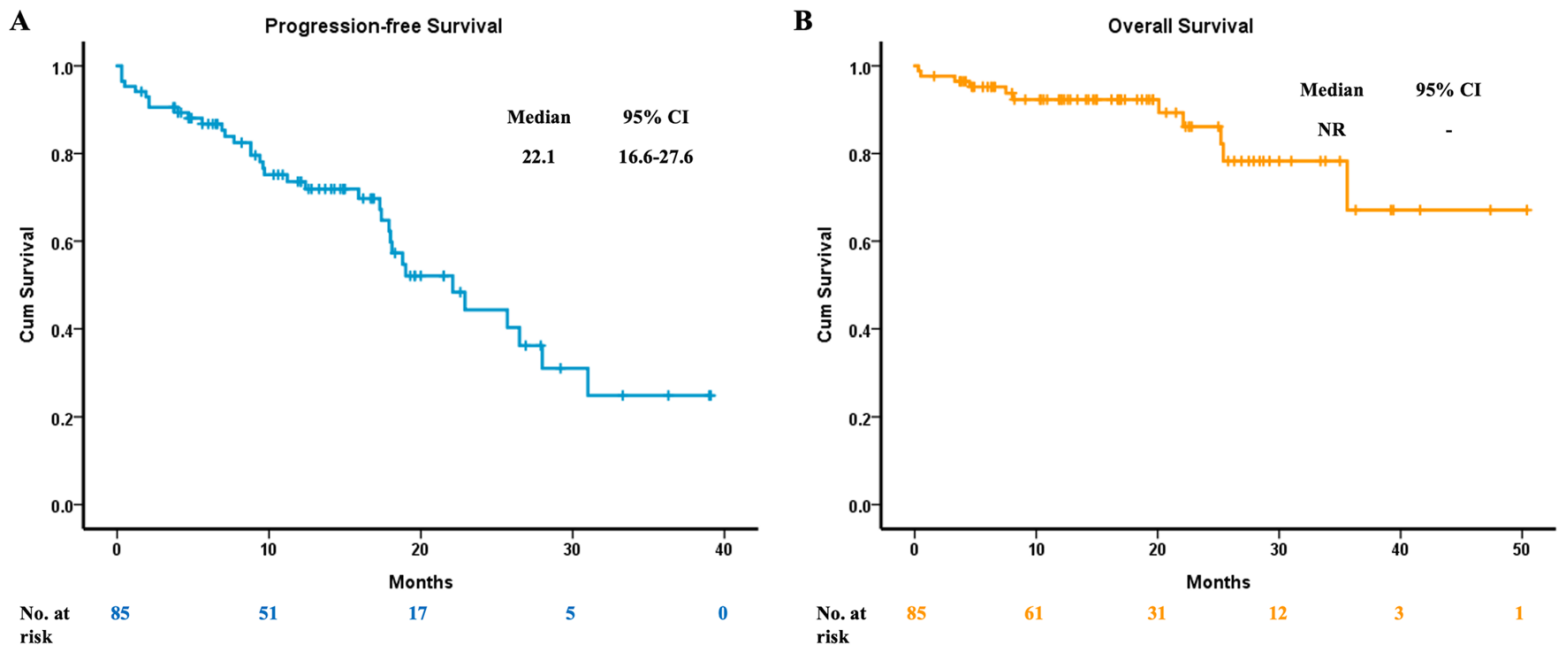
The clinical efficacy of osimertinib in advanced *EGFR*-mutant NSCLC patients. (**A**) The median progression-free survival. (**B**) The median overall survival. EGFR, epidermal growth factor receptor; NSCLC, non-small-cell lung cancer

Nine patients could not be evaluated for their treatment response to osimertinib. As for the others, one (1.3%) had a complete response, while 7 (9.2%) suffered from primary resistance. Fifty-nine (77.6%) patients experienced partial response, with 9 (11.9%) having a stable disease under osimertinib. The overall ORR was 78.9%, and the Disease Control Rate (DCR) was 90.8% (Table 1). The estimated median PFS was 22.1 months (95% Confidence Interval (CI), 16.6 to 27.6) (Fig. 1A), while the estimated median OS was not reached (Fig. 1B).

### The clinical efficacy of osimertinib in patients with measured PD-L1 expression levels

**Figure 2:**
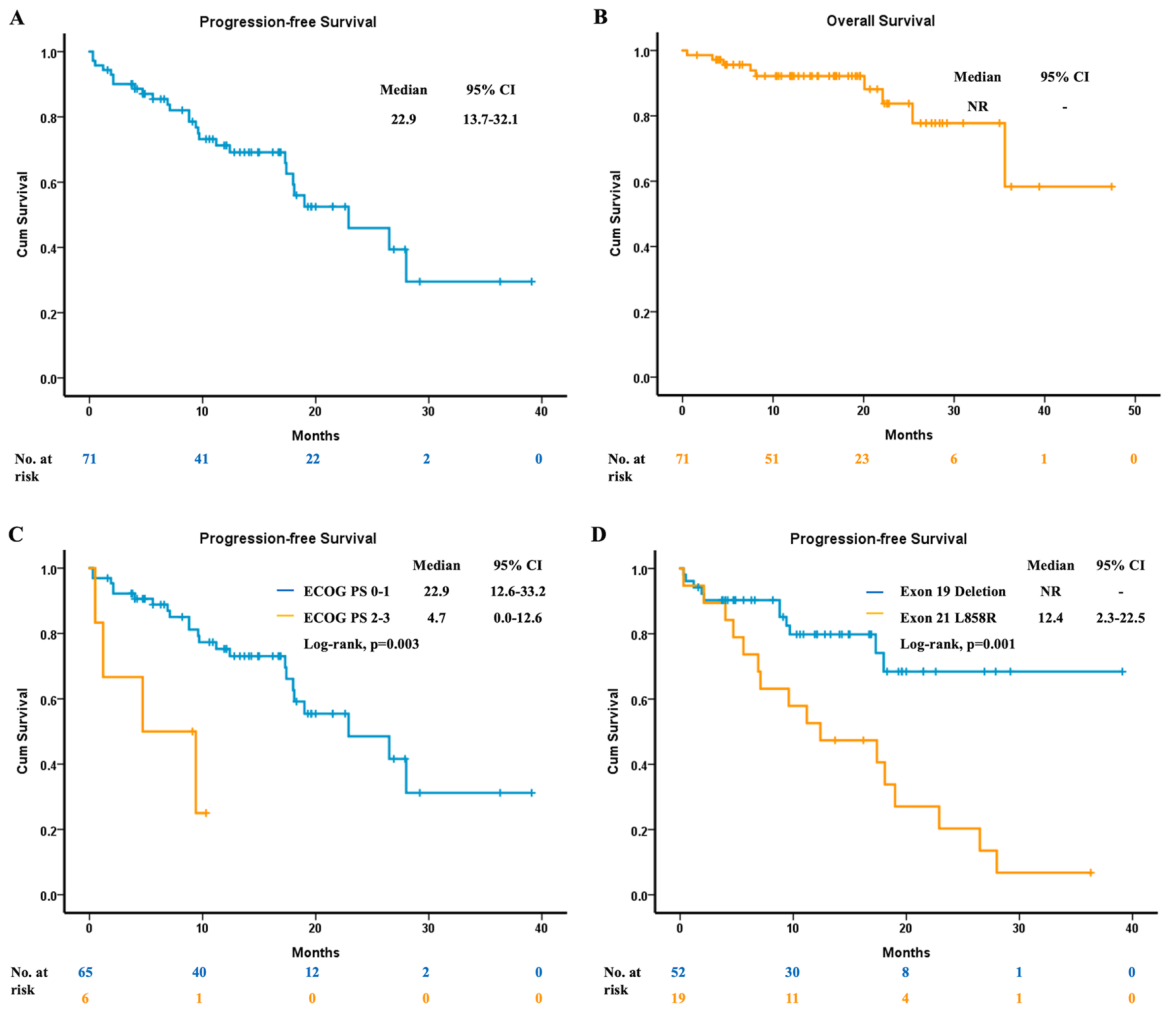
The clinical efficacy of osimertinib in patients with PD-L1 expression reports. (**A**) The median PFS. (**B**) The median OS. (**C**) The median PFS of patients with different ECOG PS. (**D**) The median PFS of patients with different baseline *EGFR* mutation status. PD-L1, programmed death-ligand 1; PFS, progression-free survival; OS, overall survival; ECOG PS, Eastern Cooperative Oncology Group performance status; EGFR, epidermal growth factor receptor.

**Figure 3:**
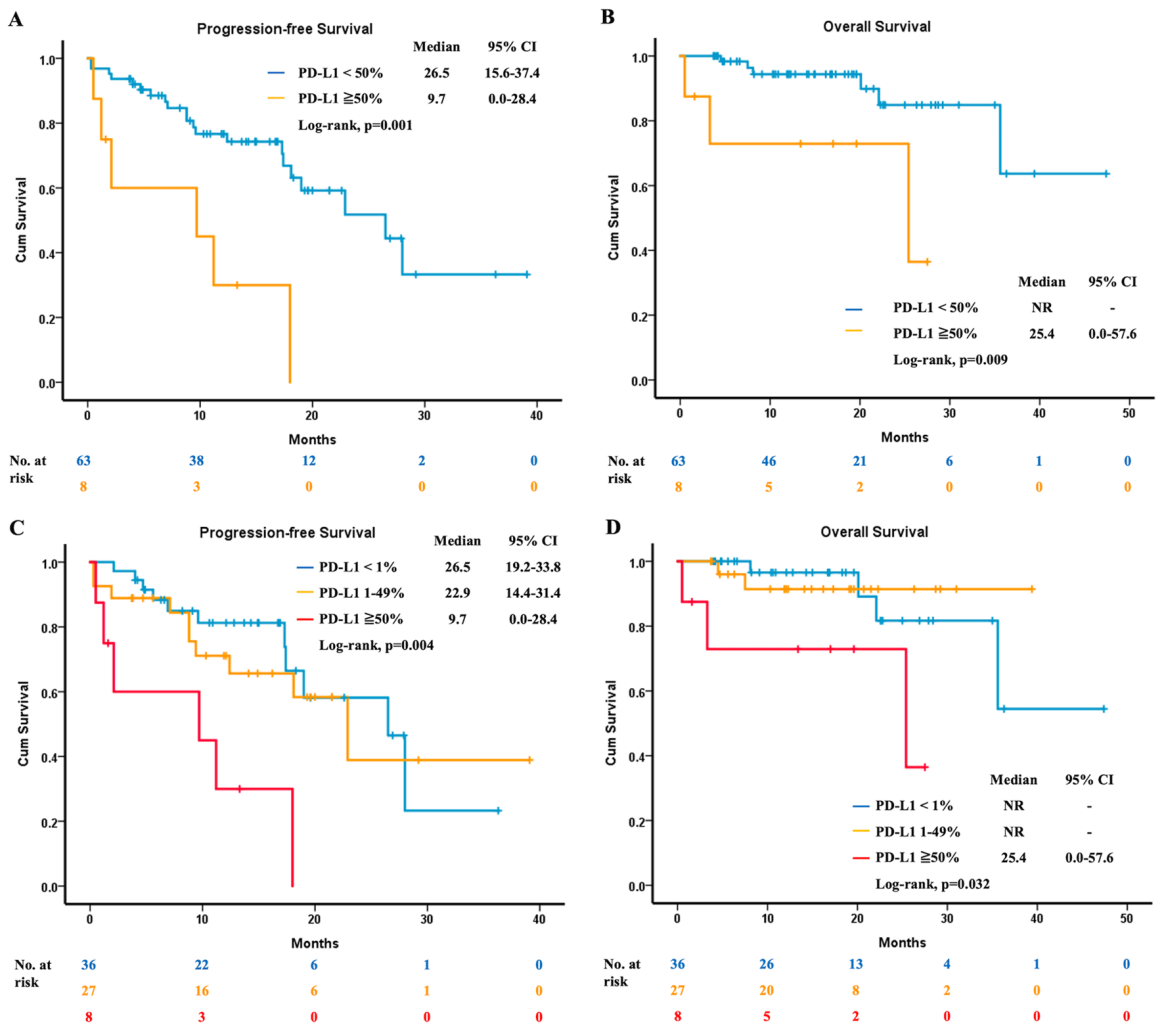
The clinical efficacy of osimertinib regarding different PD-L1 expression levels. (**A**) The median PFS in patients with PD-L1 < 50% and ≧50%. (**B**) The median OS in patients with PD-L1 < 50% and ≧50%. (**C**) The median PFS in patients with PD-L1 < 1%, 1 to 49% and ≧50%. (**D**) The median OS in patients with PD-L1 < 1%, 1 to 49% and ≧50%. PD-L1, programmed death-ligand 1; PFS, progression-free survival; OS, overall survival.

After excluding patients with unknown PD-L1 expression levels, 71 were then enrolled for analysis. Their estimated median PFS was 22.9 months (95% CI, 13.7 to 32.1) (Fig. 2A), while the estimated median OS was not reached (Fig. 2B). Concerning the different performance status, in patients with ECOG PS 0 to 1, the estimated median PFS was 22.9 months (95% CI, 12.6 to 33.2). In patients with ECOG PS 2 to 3, the estimated median PFS was 4.7 months (95% CI, 0.0 to 12.6) (Fig. 2C). Regarding different *EGFR* mutation at baseline, the estimated median PFS was not reached in patients with exon 19 deletion. In patients harboring L858R mutation, the estimated median PFS was 12.4 months (95% CI, 2.3 to 22.5) (Fig. 2D). In patients with PD-L1≧50%, the estimated median PFS was 9.7 months (95% CI, 0.0 to 28.4), and in patients with PD-L1 < 50%, the estimated median PFS was 26.5 months (95% CI, 15.6 to 37.4) (Fig. 3A). Additionally, the estimated median OS was 25.4 months (95% CI, 0.0 to 57.6) in patients with PD-L1≧50%, while in patients with PD-L1 < 50%, the estimated median OS was not reached (Fig. 3B). Furthermore, we divided all patients to three groups (PD-L1 < 1%, PD-L1 1–49% and PD-L1≧50%), and the PFS and OS were demonstrated in Fig. 3C and D, respectively.

### Univariate and multivariate analyses of PFS and OS under osimertinib in patients with measured PD-L1 expression levels

Univariate analysis revealed that patients with ECOG PS 0 to 1 had a better prognosis of PFS than patients with ECOG PS 2 to 3, where there was a Hazard Ratio (HR) of 0.21 (95% CI, 0.07 to 0.65; *p* = 0.007). Patients with PD-L1 < 50% had a statistically lower risk of progressive disease than patients with PD-L1≧50% at a HR of 0.23 (95% CI, 0.09 to 0.60; *p* = 0.002). Additionally, in patients harboring exon 19 deletion there appeared a longer PFS than there was in patients harboring L858R point mutation at a HR of 0.30 (95% CI, 0.14 to 0.64; p = 0.002), with multivariate analysis confirming the above results. In patients with ECOG PS 0 to 1, PD-L1 < 50% and exon 19 deletion had respectively better PFS with an adjusted HR (aHR) of 0.14 (95% CI, 0.04 to 0.50; p = 0.002), an aHR of 0.19 (95% CI, 0.06 to 0.67; p = 0.009) and an aHR of 0.24 (95% CI, 0.10 to 0.57; p = 0.001). (Table 2).

**Table 2:** Univariate and multivariate analyses of progression-free survival in NSCLC patients with osimertinib treatment (n = 71).

Characteristics	HR (95% CI)*	P value	Adjusted HR (95% CI)*	P value
**Age**				
< 65 ≥ 65	Reference 1.49 (0.68–3.24)	0.316	1.61 (0.68–3.78)	0.276
**Gender**				
Female Male	Reference 0.99 (0.46–2.12)	0.969	0.67 (0.14–3.07)	0.602
**Smoking status**				
C/FS NS	Reference 1.46 (0.62–3.46)	0.391	1.07 (0.22–5.35)	0.931
**ECOG PS**				
2–3 0–1	Reference 0.21 (0.07–0.65)	0.007	0.14 (0.04–0.50)	0.002
**Stage**				
Recurrence Stage IVA Stage IVB	Reference 0.90 (0.24-3.41) 1.58 (0.45–5.47)	0.871 0.474	1.09 (0.26–4.53) 1.45 (0.27–7.68)	0.909 0.665
**Brain metastasis at baseline**				
No Yes	Reference 1.92 (0.89–4.14)	0.095	1.16 (0.29–4.63)	0.834
**Baseline** ***EGFR* mutation status**				
Exon 21 L858R Exon 19 deletion	Reference 0.30 (0.14–0.64)	0.002	0.24 (0.10–0.57)	0.001
**PD-L1 expression level**				
PD-L1≧50% PD-L1 < 50%	Reference 0.23 (0.09–0.60)	0.002	0.19 (0.06–0.67)	0.009

NSCLC, non-small-cell lung cancer; HR, hazard ratio; CI, confidence interval; C/FS, current/former-smokers; NS, never smoker; ECOG PS, Eastern Cooperative Oncology Group performance status; EGFR, epidermal growth factor receptor; PD-L1, Programmed death-ligand 1.

*By Cox proportional hazard model.

Concerning OS, when compared to patients with ECOG PS 2–3, patients with ECOG PS 0–1 had a lower risk of death with an HR of 0.10 (95% CI, 0.02 to 0.46; p = 0.003), with multivariate analysis proving the result with an aHR of 0.14 (95% CI, 0.04 to 0.50; p = 0.002). Regarding PD-L1 expression levels, patients with PD-L1 < 50% had a better outcome of median OS than patients with PD-L1≧50%, with an HR of 0.18 (95% CI, 0.04 to 0.76; p = 0.020) and an aHR of 0.09 (95% CI, 0.01 to 0.70; *p* = 0.021). Additionally, patients with brain metastasis experienced worse median OS than those patients without brain metastasis at baseline, and the result was confirmed by multivariate analysis. (Table 3).

**Table 3:** Univariate and multivariate analyses of overall survival in NSCLC patients with osimertinib treatment (n = 71).

Characteristics	HR (95% CI)*	P value	Adjusted HR (95% CI)*	P value
**Age**				
< 65 ≥ 65	Reference 1.78 (0.47–6.66)	0.395	2.03 (0.42–9.82)	0.378
**Gender**				
Female Male	Reference 0.92 (0.22–3.85)	0.907	0.64 (0.06–7.19)	0.716
**Smoking status**				
C/FS NS	Reference 2.94 (0.36–23.91)	0.314	1.91 (0.12–29.37)	0.644
**ECOG PS**				
2–3 0–1	Reference 0.10 (0.02–0.46)	0.003	0.14 (0.04–0.50)	0.002
**Brain metastasis at baseline**				
No Yes	Reference 5.02 (1.01–24.85)	0.048	11.27 (1.11–114.36)	0.040
**Baseline** ***EGFR* mutation status**				
Exon 21 L858R Exon 19 deletion	Reference 0.32 (0.08–1.34)	0.118	0.08 (0.01–1.09)	0.058
**PD-L1 expression level**				
PD-L1≧50% PD-L1 < 50%	Reference 0.18 (0.04–0.76)	0.020	0.09 (0.01–0.70)	0.021

NSCLC, non-small-cell lung cancer; HR, hazard ratio; CI, confidence interval; C/FS, current/former-smokers; NS, never smoker; ECOG PS, Eastern Cooperative Oncology Group performance status; EGFR, epidermal growth factor receptor; PD-L1, Programmed death-ligand 1.

*By Cox proportional hazard model.

### Comparisons of patient characteristics between the PD-L1≧50% and PD-L1 < 50% groups

Patient baseline characteristics between the PD-L1≧50% (n = 8) and PD-L1 < 50% groups (n = 63) are compared in Table 4. In the PD-L1≧50% group, 4 (50.0%) patients were ≧65 years, while in the PD-L1 < 50% group, 25 (39.7%) patients were ≧65 years. In the PD-L1≧50% group, 4 (50.0%) patients were male, and in the PD-L1 < 50%, 25 (39.7%) were male. Most patients were nonsmokers in both groups (6 (75.0%) in the PD-L1≧50% group, and 42 (66.7%) in the PD-L1 < 50% group). In the PD-L1≧50% group, 6 (75.0%) had ECOG PS 0 to 1,while in the PD-L1 < 50% group, 59 (93.7%) patients had ECOG PS 0 to 1. Three (37.5%) patients with PD-L1≧50% had brain metastasis at the baseline, and similarly 20 (31.7%) patients in the PD-L1 < 50% group did as well. Regarding baseline *EGFR* mutation status, 6 (75.0%) patients harbored the exon 19 deletion mutation in the PD-L1≧50% group, while similarly 46 (73.0%) patients in the PD-L1 < 50% group did as well. Based on univariate analysis, no significant differences were found between the PD-L1≧50% and PD-L1 < 50% groups in age, gender, smoking status, ECOG PS, clinical stage, brain metastasis at baseline or baseline *EGFR* mutation status.

**Table 4:** The patients’ characteristics in different groups of PD-L1 expression levels (n = 71).

Characteristics	PD-L1 expression level		P value*
	≧50%, n (%)	< 50%, n (%)	
**Age**			0.708
≧65	4 (50.0)	25 (39.7)	
< 65	4 (50.0)	38 (60.3)	
**Gender, n (%)**			0.708
Male	4 (50.0)	25 (39.7)	
Female	4 (50.0)	38 (60.3)	
**Smoking status, n (%)**			1.000
Never-smokers	6 (75.0)	42 (66.7)	
Former or current-smokers	2 (25.0)	21 (33.3)	
**ECOG PS, n (%)**			0.133
0–1	6 (75.0)	59 (93.7)	
2–3	2 (25.0)	4 (6.3)	
**Stage, n (%)**			1.000
Post-operation recurrence	1 (12.5)	10 (15.9)	
Stage 4A	3 (37.5)	24 (38.1)	
Stage 4B	4 (50.0)	29 (46.0)	
**Brain metastasis at baseline, n (%)**			0.708
Yes	3 (37.5)	20 (31.7)	
No	5 (62.5)	43 (68.3)	
**Baseline** ***EGFR*mutation status, n (%)**			1.000
Exon 19 deletion	6 (75.0)	46 (73.0)	
Exon 21 L858R	2 (25.0)	17 (27.0)	

PD-L1, Programmed death-ligand 1; ECOG PS, Eastern Cooperative Oncology Group performance status; EGFR, epidermal growth factor receptor.

*By Fisher's exact test.

## Discussion

Our research in real-world practice has demonstrated that osimertinib provided satisfactory efficacy in advanced NSCLC patients harboring *EGFR* mutation. PD-L1 expression levels and ECOG PS influenced clinical outcomes of osimertinib when used as first-line treatment. Advanced *EGFR* mutated NSCLC patients with PD-L1≧50% had poorer PFS and OS than those with PD-L1 < 50%.

In the clinical trial of FLAURA on osimertinib used for patients with *EGFR*-positive NSCLC, ORR was 80% and DCR was 97%^12^. The median PFS of osimertinib was 18.9 months (95% CI,15.2 to 21.4), while the median OS was 31.8 months (95% CI, 26.6 to 36.0)^11,12^. In our present study, ORR was 78.9%, and the DCR 90.8% in advanced *EGFR*-mutant NSCLC patients having osimertinib used as their first-line treatment. The median PFS was 22.1 months (95% CI, 16.6 to 27.6), while the median OS was not reached. Our results are consistent with the results of FLAURA, confirming the clinical efficacy of osimertinib in real-world practice.

In our study, amongst the 85 patients using osimertinib as first-line treatment, 71 had PD-L1 expression reports. Thirty-six (50.7%) patients had negative results of PD-L1 expression (PD-L1 < 1%), 27 (38.0%) had weak PD-L1 expression (1–49%), and 8 (11.3%) had strong PD-L1 expression (≧50%). Previous studies have demonstrated that lung adenocarcinoma patients showed a 49.6 to 62.7% negative PD-L1 expression, 25.2 to 34.3% weak PD-L1 expressions, and 11.8 to 17.6% strong PD-L1 expressions^20,21,28^. The distribution of PD-L1 expression levels in our study was similar to those reported in the available literature.

Regarding PD-L1 expression levels and the clinical efficacy of EGFR-TKIs, Su et al., reported that strong PD-L1 expressions significantly lowered ORR, compared with the ORR of weak or negative PD-L1 expressions (35.7% versus 63.2% versus 67.3%, respectively, *p* = 0.002), while also shortening PFS (3.8 versus 6.0 versus 9.5 months, *p* < 0.001) in advanced NSCLC patients receiving EGFR-TKIs^22^. Yang et al., found that the ORR and PFS of EGFR-TKIs were both better in *EGFR* positive lung adenocarcinoma patients with PD-L1 expressions < 50%^20^. The ORR were 65.6%, 56.4% and 38.9% in PD-L1 0% group, 1 to 49% group and ≧50% group, respectively (*p* < 0.05). The PFS were 12.5, 12.8 and 5.9 months, among PD-L1 0% group, 1 to 49% group and ≧50% group, respectively (*p* < 0.05). Kang et al*.*, also showed that *EGFR*-mutant NSCLC patients with strong PD-L1 expressions had significantly shorter median PFS to EGFR-TKIs (7.07 months) than patients with weak (14.73 months, *p* < 0.001) or negative (12.70 months, *p* = 0.001) expressions^21^. A recent meta-analysis concluded that PD-L1 expression is likely a predictive biomarker for EGFR-TKI in *EGFR*-mutant NSCLC patients^29^.

Furthermore, our previous study showed that PD-L1 expression level was associated with the frequency of primary resistance to EGFR-TKIs in treatment naïve advanced lung adenocarcinoma patients with *EGFR* mutation^23^. Patients with a PD-L1≧1% experience a higher incidence of primary resistance to EGFR-TKIs than those with a PD-L1 < 1% (Odds Ratio (OR), 5.95; *p* < 0.001). This phenomenon persisted while the cutoff value was changed to either 25% (OR, 11.96; *p* = 0.001) or 50% (OR, 16.47; p = 0.008). In the study performed by Su et al., patients with de novo resistance had significantly higher PD-L1 positive rate than those with an acquired resistance to EGFR-TKIs (66.7% versus 30.2%, *p* = 0.009)^22^. Yang et al*.*, demonstrated that *EGFR*-mutant lung adenocarcinoma patients with PD-L1≧50% had a higher chance of primary resistance to EGFR-TKI than patients with PD-L1 1 to 49% and patients with PD-L1 < 1% (44.4% versus 2.6% versus 6.3%, respectively; p < 0.001)^20^. Kang et al*.*, also found that early progression rates, defined as progressive disease occurring within 6 months after first-line EGFR-TKI treatment had begun, were 12.7%, 2.7%, and 37.5% in patients with negative, weak, and strong PD-L1 expression, respectively (p = 0.004)^21^. Based on the above findings, not only PFS but also the chance of de novo resistance to first-line EGFR-TKIs, were correlated with PD-L1 expressions in *EGFR*-mutant NSCLC patients.

Although previous research has discussed the relationship between PD-L1 expression levels and the clinical efficacy of EGFR-TKIs, there have been few papers focusing on the third-generation EGFR-TKI osimertinib. In the FLAURA study, post-hoc analysis showed that in advanced treatment naïve *EGFR*-mutant NSCLC patients with PD-L1≧1%, the median PFS was 18.4 months with osimertinib, and 6.9 months with gefitinib and erlotinib (HR 0.30 (95% CI, 0.15 to 0.60))^24^. Among patients with PD-L1 negative, the median PFS of osimertinib was 18.9 months, and 10.9 months in patients receiving gefitinib and erlotinib (HR 0.37 (95% CI, 0.17 to 0.74)). The ORR to osimertinib in patients with PD-L1≧1% was 79%, while in patients with PD-L1 < 1% it was 85%. Brown et al., concluded that the clinical outcomes of first-line osimertinib treatment for advanced *EGFR*-mutant NSCLC was not affected by PD-L1 expression status. However, these authors did not analyze the impact of strong PD-L1 expression (≧50%) on the clinical efficacy of osimertinib. In our present study, we found that advanced *EGFR*-mutant NSCLC patients with PD-L1 < 50% experienced a longer PFS than those with PD-L1≧50% (26.5 versus 9.7 months, respectively, aHR 0.19 (95% CI, 0.06 to 0.67); p = 0.009). Patients with PD-L1 < 50% had better OS than patients with PD-L1≧50% (NR versus 25.4 months, respectively, aHR 0.09 (95% CI, 0.01 to 0.70); p = 0.021). Strong PD-L1 expressions resulted in statistically significant poorer prognoses in patients being treated with osimertinib as their first-line EGFR-TKI treatment.

Previous studies have demonstrated several mechanisms which could result in primary resistance to EGFR-TKI in *EGFR*-mutant NSCLC patients. These mechanisms include, Phosphatase and Tensin homolog gene (PTEN) loss, Erb-b2 receptor tyrosine kinase 2 gene (ERBB2) amplification*, *de novo T790M mutation, *MET* amplification, v-myc avian myelocytomatosis viral oncogene homolog gene (MYC) amplification, BIM deletion polymorphism, and overexpression of CRIPTO1^30–32^. Previous studies have also reported that *EGFR* activation upregulated PD-L1 expression through the p-ERK1/2/p–c-Jun pathway, while *MET* activation also promoted the expression of PD-L1^33,34^. In Kang’s study, strong PD-L1 expressions in *EGFR* positive NSCLC specimens were related to both the JAK-STAT pathway and *MUC16* mutation frequency^21^. The authors found that the activation of the JAK-STAT pathway may play the role of a de novo resistance mechanism to EGFR-TKIs^21^. The above findings may explain the mechanisms surrounding drug resistance to EGFR-TKIs in *EGFR*-mutant NSCLC patients with strong PD-L1 expressions. Additional studies are still needed in order to obtain a complete understanding of the mechanisms involved. In clinical practice, we should notice that NSCLC patients with *EGFR*-mutant and high PD-L1 expression may experience relatively poor outcomes than patients with PD-L1 < 50%. In the future, we should figure out the possible treatment strategies for these patients. Combination treatment with anti-angiogenic agents or chemotherapy may overcome the downside. However, we need clinical trials to confirm our idea.

There were certain limitations to our study. First, it was a single center, retrospective study with inevitable bias, as compared with prospective studies. Second, only Taiwanese subjects were studied. Therefore, our findings may not be generalized to include other ethnic populations. Third, osimertinib treatment patients began to be reimbursed for their treatment costs in April 2020. For this economic reason, many patients did not receive osimertinib treatment prior to this time, accounting for the relatively small number of patients enrolled for our analysis. Fourth, the follow-up time period was not long enough, with the maturation rate of PFS reaching only 41.2% and the OS measuring 12.9%. Fifth, not all patients given osimertinib as first-line EGFR-TKI treatment could provide data regarding PD-L1 expression. Finally, PD-L1 expression was determined using two different kits (Ventana SP263 and Dako 22C3). However, the Blueprint project had previously reported that three PD-L1 IHC assays (Ventana SP263, Dako 22C3 and Dako 28-8) were closely aligned in their results which involved tumor cell staining^35^. Thus, we believe that the different kits we used did not influence our results.

In conclusion, our study has demonstrated that patients receiving osimertinib as first-line treatment had satisfactory ORR and PFS. Of particular note, patients with strong PD-L1 expression displayed poor outcomes after osimertinib treatment in terms of both PFS and OS. We still need multi-center retrospective studies or clinical trials to confirm our finding.

## Data Availability

The datasets generated during and/or analysed during the current study are not publicly available due to patients’ privacy but are available from the corresponding author on reasonable request.
